# Genome-Wide Identification and Validation of Gene Expression Biomarkers in the Diagnosis of Ovarian Serous Cystadenocarcinoma

**DOI:** 10.3390/cancers14153764

**Published:** 2022-08-02

**Authors:** Francesca Zalfa, Maria Grazia Perrone, Savina Ferorelli, Luna Laera, Ciro Leonardo Pierri, Anna Tolomeo, Vincenzo Dimiccoli, Giuseppe Perrone, Anna De Grassi, Antonio Scilimati

**Affiliations:** 1 Predictive Molecular Diagnostic Unit, Pathology Department, Fondazione Policlinico Universitario Campus Bio-Medico, 00128 Rome, Italy; f.zalfa@policlinicocampus.it; 2 Microscopic and Ultrastructural Anatomy Unit, CIR, Fondazione Policlinico Universitario Campus Bio-Medico, 00128 Rome, Italy; 3 Department of Pharmacy-Pharmaceutical Sciences, University of Bari “Aldo Moro”, 70125 Bari, Italy; mariagrazia.perrone@uniba.it (M.G.P.); savina.ferorelli@uniba.it (S.F.); 4 Department of Biosciences, Biotechnologies, Biopharmaceutics, University of Bari “Aldo Moro”, 70125 Bari, Italy; laeraluna@gmail.com (L.L.); ciro.pierri@uniba.it (C.L.P.); 5 Department of ITELPHARMA, ITEL Telecomunicazioni S.R.L., 70037 Ruvo di Puglia, Italy; a.tolomeo@itelte.it (A.T.); v.dimiccoli@itelte.it (V.D.); 6 Pathology Department, Fondazione Policlinico Universitario Campus Bio-Medico, 00128 Rome, Italy; g.perrone@policlinicocampus.it; 7 Pathology Research Unit, Fondazione Policlinico Universitario Campus Bio-Medico, 00128 Rome, Italy

**Keywords:** ovarian serous cystadenocarcinoma (OSCA), biomarkers, TCGA transcriptomes, digital RNA detection, NanoString technology

## Abstract

**Simple Summary:**

Despite ovarian serous adenocarcinoma (OSCA) is a high-incidence type of cancer, limited molecular screening methods are available and the diagnosis mostly occurs at a late stage. The aim of this study is screening the potential of gene expression for identifying OSCA-specific molecular biomarkers for improving diagnosis. A genome-wide survey was performed on high-throughput RNA-sequencing experiments on hundreds ovarian cancer samples and healthy ovarian tissues, providing a number of putative OSCA biomarkers, which were then validated on an independent sample set and using a different RNA-quantification technology. Combinations of gene expression biomarkers were identified, which showed high accuracy in discriminating OSCA tissues from the normal counterpart and from other tumor types. The detected biomarkers can improve the molecular diagnosis of OSCA on tissue samples and are, in principle, translatable to the analysis of liquid biopsies.

**Abstract:**

Ovarian cancer is the second most prevalent gynecologic malignancy, and ovarian serous cystadenocarcinoma (OSCA) is the most common and lethal subtype of ovarian cancer. Current screening methods have strong limits on early detection, and the majority of OSCA patients relapse. In this work, we developed and cross-validated a method for detecting gene expression biomarkers able to discriminate OSCA tissues from healthy ovarian tissues and other cancer types with high accuracy. A preliminary ranking-based approach was applied, resulting in a panel of 41 over-expressed genes in OSCA. The RNA quantity gene expression of the 41 selected genes was then cross-validated by using NanoString nCounter technology. Moreover, we showed that the RNA quantity of eight genes (*ADGRG1*, *EPCAM*, *ESRP1*, *MAL2*, *MYH14*, *PRSS8*, *ST14* and *WFDC2*) discriminates each OSCA sample from each healthy sample in our data set with sensitivity of 100% and specificity of 100%. For the other three genes (*MUC16*, *PAX8* and *SOX17*) in combination, their RNA quantity may distinguish OSCA from other 29 tumor types.

## 1. Introduction

Ovarian cancer (OC) is one of the most lethal gynecological cancers. It is the seventh leading cause of cancer-related death among women. In the years 2008–2016, there was an increment of almost ten thousand deaths per year [1,2,3,4]. The high mortality rate is caused by a late-stage diagnosis, mainly because OC has unspecific symptoms in disease onset, and no diagnostic procedures with adequate sensitivity and specificity suitable for precocious screening methods are yet available. The identification of tumor-selective and -specific biomarkers is necessary for obtaining an early OC diagnosis, improving patient prognoses and reducing mortality.

Nearly 80% of OC cases occur in women aged 20–75 years, and less than 5% occur in those of pediatric age [5,6,7]. The incidence of OC varies by geographic area, with a higher incidence rate in the United States and Europe (15 new OCs/100 thousand women) than in Asia and the Middle East (2 new OCs/100 thousand women) [1]. In the last decade, the prevalence of OC was around 2%, and the median survival rate (SR) was ~30% at 5 years after diagnosis. These data are almost unchanged over the last 50 years. Such a low SR mainly depends on the fact that most diagnoses are made at very late stages of OC [8] because a screening gold standard or biomarker for the early detection of the disease has not yet been established [9]. However, only 20% of ovarian cancer cases can be detected at stage I or II [International Federation of Gynecology and Obstetrics (FIGO)] by conventional gynecologic examination combined with the measurement of carbohydrate antigen 125 (CA125) in serum and transvaginal ultrasound. In this case, the prognoses of these patients are generally good, with a 5-year overall survival rate of up to 90%. The false-positive rate of CA125 is approximately 50% with iatrogenic morbidity due to unnecessary surgery [10,11]. The predictivity of CA125 is approximately 10%, reaching 20% when coupled with transvaginal ultrasound and the recent FDA-approved quantification of serum biomarker HE4 (human epididymis protein) [12,13]. However, it must be considered that CA125 and HE4 proteins are neither gender nor tumor-specific.

According to the histological classification, OC can be epithelial, stromal or germinal. Epithelial ovarian cancer (EOC) is the most common OC type (90–95%) and has a high degree of heterogeneity [14,15]. It is, in fact, further subclassified as serous (70% of EOC), mucinous, endometroid, clear cell, transitional cell, squamous cell, mixed epithelial and undifferentiated [16,17,18]. Serous EOC has four subtypes (mesenchymal, immunoreactive, differentiated and proliferative) and is increasingly diagnosed as high-grade serous ovarian cancer (HGSOC), also called ovarian serous cystadenocarcinoma (OSCA) [19,20,21]. Each histological subtype is associated with a distinct clinical outcome of the disease [response to chemotherapy, pattern of metastases, PFS and overall survival (OS)], even though it is treated as one entity, with very few exceptions.

Genome-wide expression profiling investigations on EOCs of various histologies have elucidated both the distinguishable global gene expression profiles and the signaling pathways that contribute to the biological and clinicopathological characteristics of the four major subtypes, although some overlap exists [22]. Distinct genomic abnormalities (gene amplification, deletion and mutation) exist among the various OC subtypes [23].

Today, no true diagnostic or predictive screening tests are available. An analysis of gene expression data derived from The Cancer Genome Atlas (TCGA) study of high-grade ovarian serous carcinoma [HGSOC, equivalent to TCGA Ovarian Serous Cystadenocarcinoma (OSCA)] indicated that individual tumor samples could have multiple subtype signatures with different levels of activation. Most samples have a dominant signature that is stable across tumor deposits [3,21]. Therefore, a comprehensive genetic characterization of a large number of OCs is necessary, and the impact of the molecular subset on drug sensitivity and patient outcome can be determined.

Here, we report a bioinformatic re-analysis of data reported in the TGCA and GTEx portal in an attempt to identify putative OSCA biomarkers for possible use in general screening and/or diagnosis. The result of this analysis was validated through NanoString technology by analyzing transcripts from forty-two human OSCA samples, which were then compared to those from normal ovarian tissues.

## 2. Results

### 2.1. Identification of 41 Putative Gene Expression OSCA Biomarkers by Transcriptome Analyses

**Figure 1:**
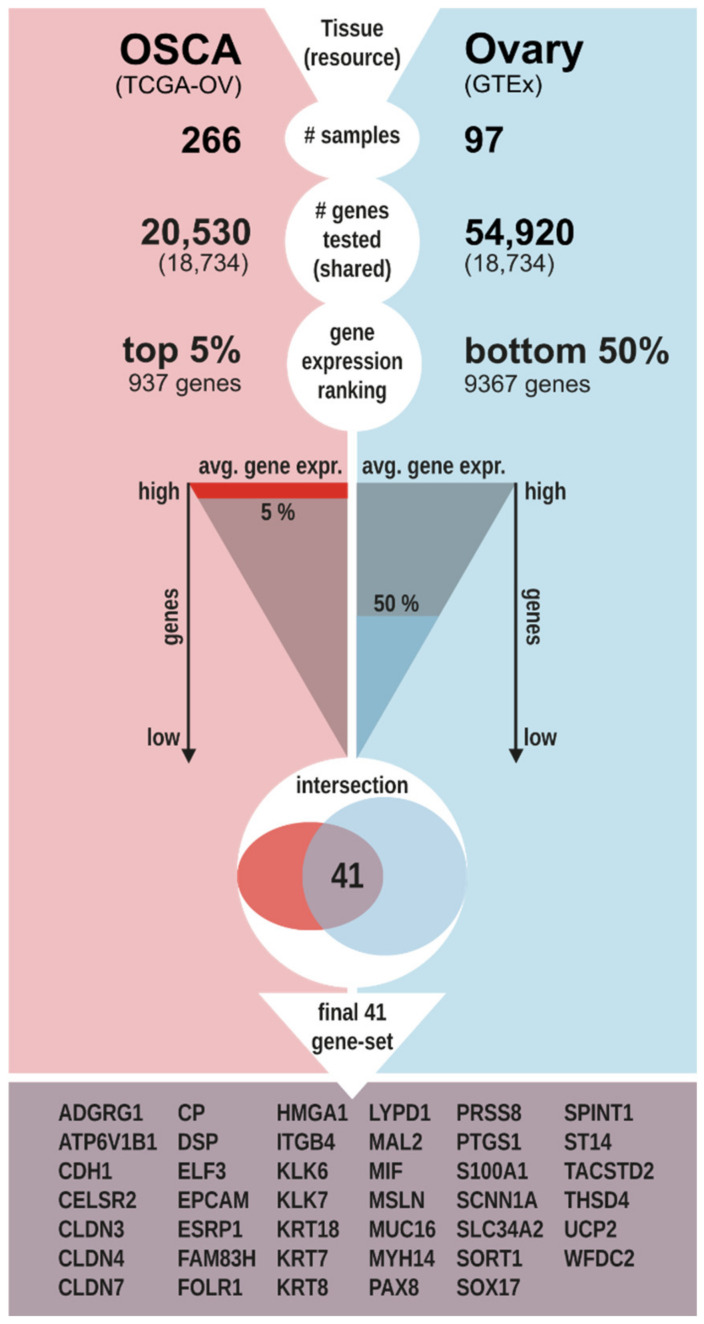
Biomarker identification pipeline. The workflow was applied to identify the 41 putative OSCA gene expression biomarkers in OSCA tissues compared to healthy ovarian tissues by gene expression ranking analysis of RNA-seq transcriptomes recovered from TCGA and GTEx.

To find genes that over-express in OSCA compared to the healthy ovary, two independent RNA-seq-based transcriptomes were screened, hosting 266 OSCA tissues from TCGA and 97 healthy ovarian tissues (control) from GTEx, respectively. Since different protocols for the RNA quantification and genome annotation of the two original datasets prevented direct comparison of gene expression profiles, 18,734 genes were identified as analyzed in both the data sets and were ranked by gene expression values in each data set, separately. The ranking analysis resulted in 937 genes showing the highest 5% average expression values among OSCA samples and 9367 genes showing the 50% lowest average expression values among control samples. A subset of 41 genes passed both criteria (Figure 1 and Supplementary Table S1), and they were consequently predicted to be over-expressed in OSCA compared to the control and are hereinafter referred to as putative biomarkers. A Gene Ontology enrichment analysis showed that the 41 putative biomarkers are significantly enriched in proteins involved in epithelium developmental processes and components of extracellular vesicles (Supplementary Figure S1).

### 2.2. Biomarker Validation by Changes in OSCA Samples and RNA Quantification Technology

**Figure 2:**
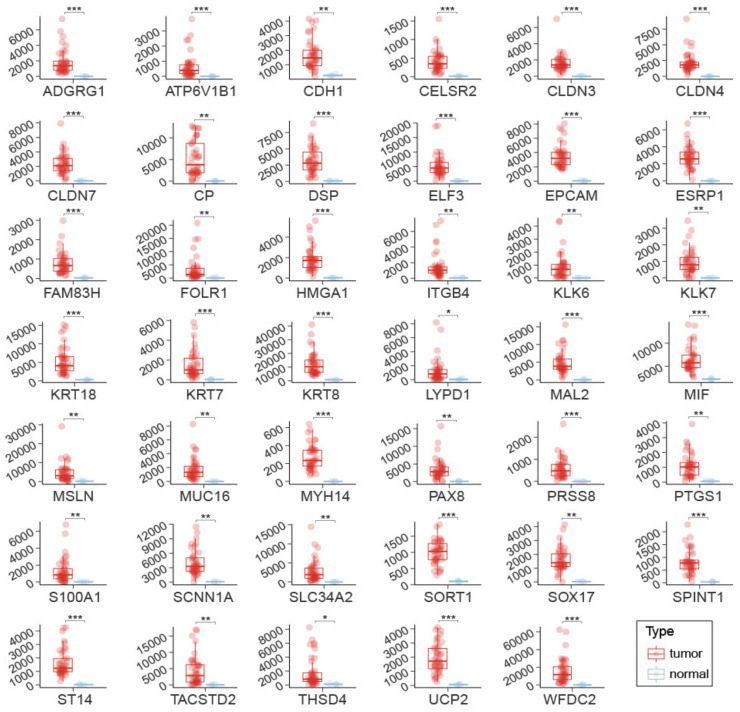
RNA quantification of the 41 putative biomarkers using NanoString nCounter. Gene expression is reported for each of the 41 genes in each of the 42 OSCA tissues (red dots and box plots) and the 3 healthy ovarian tissues (blu dots and box plots). Boxes indicate parameters of RNA quantity distribution (1st quartile, median and 3rd quartile). The statistical significance of the difference in gene expression between the tumor and normal samples was calculated using a non-parametric two-tailed Wilcoxon test (*, *p* < 0.05; **, *p* < 0.01, ***, *p* < 0.001).

To test the robustness of the 41 biomarker genes, their expression was measured on an independent sample set (Supplementary Table S2), i.e., 42 OSCA tissues and 3 healthy ovarian tissues (control), using the innovative gene expression analysis technology NanoString nCounter that allows the direct quantification of RNA molecules, in contrast to the RNA-seq technology that requires preliminary steps of reverse transcription of RNA into cDNA and the amplification of the cDNA library before sequencing. Despite the low number of normal samples and high RNA quantity variability across tumor samples, each of the 41 putative biomarkers showed statistically significant higher RNA quantity in OSCA tissues compared to the control (Figure 2). These results validated the ranking-based method applied to the analysis of transcriptomes and fostered downstream analyses for all the 41 putative biomarkers.

**Figure 3:**
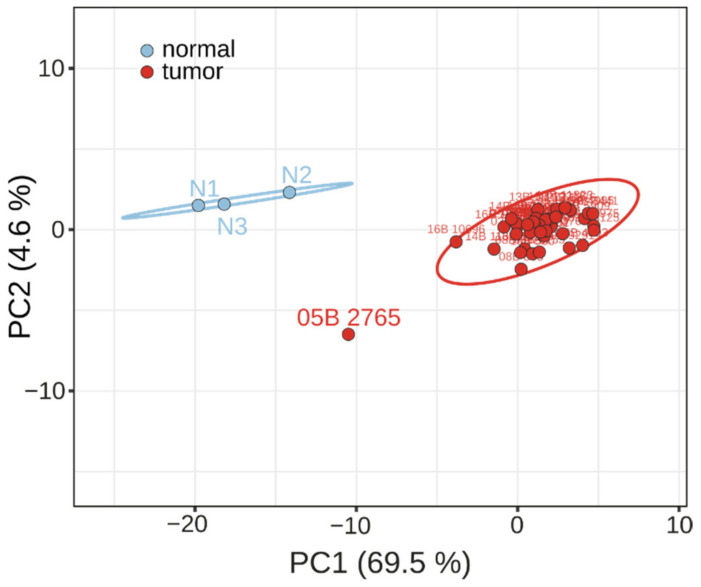
PCA analysis of RNA expression. Dots indicate OSCA tissue samples (red) and normal ovary tissue samples (blu). X and Y axis show principal component 1 (PC1) and principal component 2 (PC2), which explain 69.5% and 4.6% of the total variance, respectively. Ellipses indicate that a new sample from the same tissue type will fall inside the ellipse with a probability 0.95.

A PCA analysis was conducted to assess whether the NanoString-based gene expression profile of the 41 genes was sufficient to correctly identify OSCA samples from control samples. The PCA results showed that all the samples but one group in two separated clusters correctly matched the corresponding tissue type (Figure 3). Regarding the outlier OSCA sample (ID 05B 2765), falling outside both clusters, a re-examination of the patient’s medical history revealed that she had been surgically and pharmacologically treated for breast cancer before the ovarian cancer diagnosis and had been again surgically treated for breast cancer afterward, raising doubts about the origin and type of this tissue. The PCA results allowed two further considerations. First, the probability of additional samples being correctly classified as OSCA is 95%, confirming the robustness of the 41-gene panel. Second, the PCA X axis explains most of the total variance (69.5%), suggesting that less than 41 genes may be sufficient to correctly discriminate OSCA samples from controls.

### 2.3. Discriminating Power of Individual OSCA Biomarker Genes

**Figure 4:**
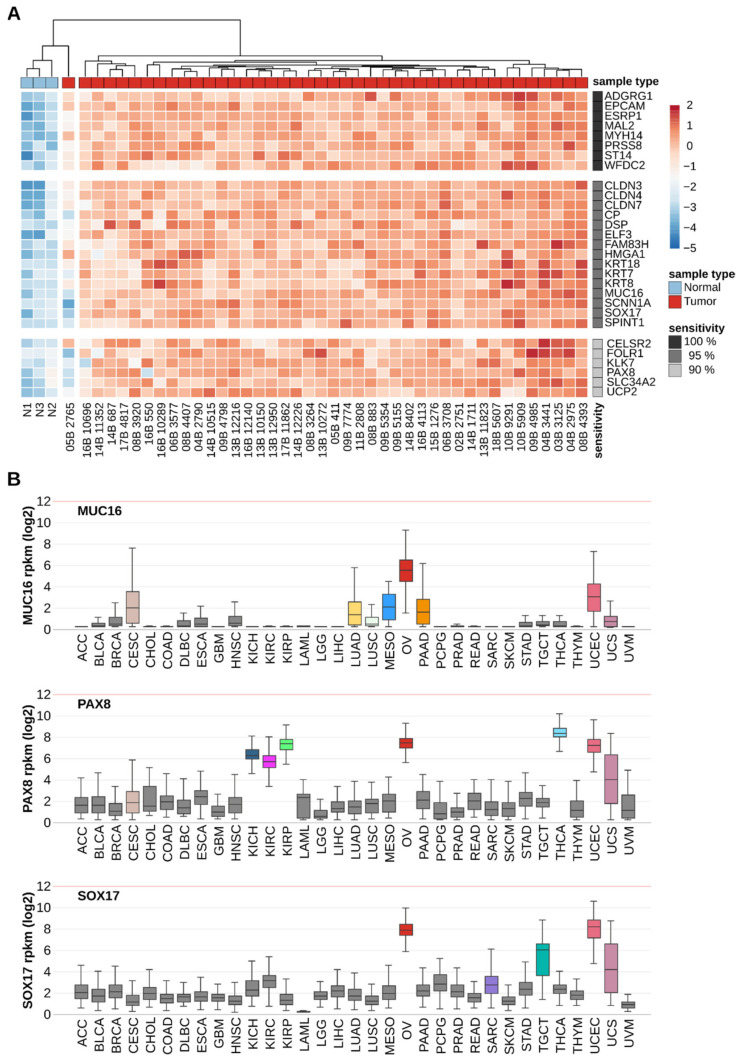
Discriminating power of single OSCA biomarker genes. (**A**) HeatMap of gene expression levels derived from the NanoString nCounter analysis for 29 genes showing 100% specificity and high sensitivity (100%, 95%, 90%) across 42 OSCA samples (tumor) and 3 healthy ovarian samples (normal). (**B**) Box plots of the gene expression distributions across 11,003 samples from 33 TCGA tumor types. Colors indicate OSCA (red), tumor types with distributions that do not overlap with that of OSCA (gray) or that do overlap with that of OSCA (other colors). ACC: Adrenocortical carcinoma; BLCA: Bladder Urothelial Carcinoma; BRCA: Breast invasive carcinoma; CESC: Cervical squamous cell carcinoma and endocervical adenocarcinoma; CHOL: Cholangiocarcinoma; COAD: Colon adenocarcinoma; DLBC: Lymphoid Neoplasm Diffuse Large B-cell Lymphoma; ESCA: Esophageal carcinoma; GBM: Glioblastoma multiforme; HNSC: Head and Neck squamous cell carcinoma; KICH: Kidney Chromophobe; KIRC: Kidney renal clear cell carcinoma: KIRP: Kidney renal papillary cell carcinoma; LAML: Acute Myeloid Leukemia; LGG: Brain Lower Grade Glioma; LIHC: Liver hepatocellular carcinoma; LUAD: Lung adenocarcinoma; LUSC: Lung squamous cell carcinoma; MESO: Mesothelioma; OV: Ovarian serous cystadenocarcinoma; PAAD: Pancreatic adenocarcinoma; PCPG: Pheochromocytoma and Paraganglioma; PRAD: Prostate adenocarcinoma; READ: Rectum adenocarcinoma; SARC: Sarcoma; SKCM: Skin Cutaneous Melanoma; STAD Stomach adenocarcinoma; TGCT: Testicular Germ Cell Tumors; THCA: Thyroid carcinoma; THYM: Thymoma; UCEC: Uterine Corpus Endometrial Carcinoma; UCS: Uterine Carcinosarcoma; UVM: Uveal Melanoma.

The NanoString-based gene expression profile of each of the 41 genes was independently analyzed to find the best biomarkers with respect to their accuracy in discriminating between OSCA and control samples. For each gene, specificity was set to 100% by fixing an RNA-quantity threshold to 5-fold the highest value among the three control samples, and sensitivity was calculated as the percentage of OSCA samples for which the RNA quantity exceeded the fixed threshold. Despite the highly stringent threshold, a subset of 29 genes resulted in being individually sufficient to discriminate OSCA samples from the control sample with high sensitivity (Figure 4A). Specifically, 8 genes showed 100% sensitivity, as each of them correctly identified each OSCA sample, including the outlier sample 05B 2765; whereas 15 genes and 6 genes showed 95% and 90% sensitivity, respectively. The decrease in sensitivity of the latter was partially due to the outlier sample 05B 2765, which showed low expression for many genes including the well-known OSCA protein markers encoded by *MUC16* and *FOLR1* (Figure 4A).

Since the expression of the 29-gene subset demonstrated high accuracy in discriminating OSCA tissues versus healthy ovarian tissues, they were further filtered for their potential in detecting OSCA versus other tumor types to predict whether OSCA cells may be selectively distinguished from other cancer cells, in case body districts other than the ovary are screened. The gene expression analysis of the full set of 11,003 samples from 33 TCGA tumor types identified three genes (MUC16, PAX8 and SOX17) that showed gene expression ranges of OSCA samples that did not overlap with the gene expression ranges of samples deriving from most of the other tumor types (Figure 4B). Specifically, non-overlapping gene expression intervals were observed between OSCA and the other 25 tumor types for MUC16, the other 25 tumor types for PAX8 and the other 29 tumor types for SOX17, and the combination of the three genes may, in principle, discriminate OSCA from any other TCGA-screened tumor type except Uterine Corpus Endometrial Carcinoma (UCEC) and Uterine Carcinosarcoma (UCS) (Figure 4B).

## 3. Discussion

Accurate ways to detect ovarian cancer early could have a great impact on the treatment of patients and their overall survival. According to a recent study, the simultaneous detection of six biomarkers [leptin, prolactin, osteopontin, insulin-like growth factor II (IGF-II), macrophage inhibitory factor (MIF), and CA125] would have 95% sensitivity and 99% specificity for ovarian cancer, particularly higher than that of CA125 alone. Dosing this panel of antigens (OVALife test) provides a reliable screen of patients at high risk, such as women younger than 50 years old with positive familiarity for ovarian or breast cancer, particularly if they bear a BRCA1 or BRCA2 gene mutation or belong to families with Lynch 2 syndrome [23]. Further developed tests OvaSure and OvaCheck were never shown to help find OC [24,25,26,27,28]. More recently, new tests have tried to detect OC by measuring genes or proteins. In the case of the OVA1 test, five proteins are measured [CA125, apolipoprotein A1, beta-2 microglobulin, transferrin and pre-albumin [29]. OVA1 is not intended to be a screening test for OC, being FDA approved with stringent criteria in the selection of women over 18 years of age, with the presence of an ovarian adnexal mass, who have surgery planned, who have not yet been referred to a gynecologic oncologist and who have not had cancer in the past 5 years. Other tests to be performed alone and in combination are CA125 (not specific), ultrasounds of the abdomen and pelvis and Positron Emission Tomography (PET-CT). The PET radiotracer [^18^F]-FDG alone has 78% sensitivity and 68% specificity [30,31]. Further studies attempted to identify novel PET radiotracers [32] and fluorescent probes [33,34] targeting unexplored putative biomarkers.

Due to the existence of few low specific biomarkers for the early detection of OC [35,36,37,38,39,40], our investigations tried to identify an unambiguous set of biomarkers, characterized by high specificity and selectivity, suitable to screening and detecting OSCA [41].

With this aim, we retrieved and integrated data obtained by two human large-scale projects that generated transcriptomic data from 266 ovarian cancer tissues (TCGA) and 97 ovarian normal tissues (GTEx). GTEx data for ovarian non-cancer tissues were investigated due to the lack of gene expression data about ovarian cancer in TCGA. For comparing the expression data collected from the two datasets, a ranking analysis was performed [42,43,44]. It revealed that 41 genes expressed 3 to 300 times more in tumor samples than in their non-cancer counterpart. The 41 genes were introduced in a panel of genes for the following validation through NanoString analysis used for quantifying the corresponding RNA from specimens collected from 42 OSCA patients and 3 ovarian non-cancer tissues. The proposed 41-gene biomarker set was able to discriminate between OSCA and non-cancer samples with high specificity, showing that the proposed candidate biomarkers rapidly detect OSCA independently from the analyzed samples and the employed RNA quantification technology [41].

The only exception was a tumor sample noteworthy for the low expression values of many genes (see sample 05B 2765), which raises the question of a possible different metastatic origin of this sample (Figure 3). This sample belonged to a patient treated surgically and chemically for previous breast cancer. This latter observation is also particularly interesting because, with this gene set, it would be possible to distinguish patients with native ovarian cancer from metastatic ovarian cancer (i.e., ovarian cancer resulting from treated breast cancer).

## 4. Conclusions

In summary, through integrated transcriptomic analysis, a panel of 41 genes was identified that were found to be expressed 3- to 300-fold more in ovarian cancer than in non-tumor samples (Figure 2). Among these genes, there are 29 biomarker genes with high diagnostic power of OSCA compared with healthy ovarian tissues (Figure 4A), and 3 of these genes also show high diagnostic power of OSCA compared with other tumor types (Figure 4B). Since tumors, in general, release more RNA into the bloodstream than normal tissues, the biomarker genes identified in this study should also be tested for OSCA diagnostics in plasma-derived samples. Further studies are underway to increase the number of patients who are 42 and to try to validate the current diagnostic capability on liquid biopsies and/or urine. Metabolomic and metagenomic investigations of OCs have also been initiated.

## 5. Methods

### 5.1. Transcriptome Data Analysis

Data sets from high-throughput RNA sequencing experiments (RNA-seq) were recovered from two distinct resources: The Cancer Genome Atlas for OSCA samples (TCGA [45], TCGA-OV dataset, quantification as TPM and annotation to 20,530 gene identifiers) and the Genotype-Tissue Expression project for normal ovarian samples (GTEx [46], ovary dataset, quantification as RPKM and annotation to 54,920 gene identifiers). Custom Perl scripts were used to calculate and compare gene expression ranks between OSCA and normal ovary samples using the following criteria. First, the average gene expression value among samples was calculated for each gene in each data set. Second, the resulting two gene lists were separately ordered by average gene expression values from the highest to the lowest expressed gene. Third, a gene was selected as a putative biomarker if it showed the highest 5% average expression value among OSCA samples and the lowest 50% average expression value among normal ovary samples (Figure 1).

Distributions of gene expression values across 33 TCGA cancer datasets were generated using the R2 Genomics Analysis and Visualization Platform [47] using the dataset “Mixed Cancer GDC - TCGA - 11003 – rpkm”.

### 5.2. RNA Extraction and Quantification through NanoString Analysis

Total RNA was extracted using the High Pure FFPET RNA Isolation Kit (Roche) from 10 μm sections of 42 formalin-fixed paraffin-embedded (FFPE) OSCA tissues and 3 FFPE ovarian healthy tissues. RNA was quantified with the BioPhotometer^®^ D30 (Eppendorf), and 100 ng of total RNA was subjected to the NanoString nCounter Analysis System. This system utilizes a novel digital color-coded barcode technology that is based on direct multiplexed measurements of gene expression and offers high levels of precision and sensitivity (<1 copy per cell). The technology uses molecular “barcodes” and single molecule imaging to detect and count hundreds of unique transcripts in a single reaction. The nCounter CodeSet was customized with 41 genes and 6 housekeeping genes (CLTC, GAPDH, GUSB, HPRT1, PGK1 and TUBB) to normalize the expression values. NanoString nCounter analysis was performed following the manufacturer’s protocol (www.nanostring.com, accessed on 1 November 2020). In brief, 100 ng of total RNA was hybridized in solution at 65 °C for 16 h with specific pairs of ~50 base probes for each gene set of mRNA. The Reporter Probe carried the signal, and the Capture Probe allowed the complex to be immobilized for data collection. After hybridization, the excess probes were removed, and the probe/target complexes aligned and immobilized in the nCounter Cartridge. Sample Cartridges were placed into the Digital Analyzer for data collection. Color codes on the cartridge surface were counted and tabulated for each target molecule. Raw counts for each mRNA were normalized for housekeeping genes using nSolver Analysis Software (nSolver 4.0, NanoString Technologies Inc., Seattle, WA, USA).

### 5.3. Gene Ontology, PCA and Measures of Accuracy

Gene Ontology enrichment analyses were conducted using g: Profiler [48] with default parameters (g: SCS methods for multiple testing correction, *p* value < 0.05). Principal component analysis (PCA) was conducted using ClustVis [49], and the original values were ln(x)-transformed. Unit variance scaling was applied to the rows, and SVD with imputation was used to calculate the principal components (Figure 3). A HeatMap was generated by ClusVis with parameters “clustering distance: maximum” and “clustering method: complete” (Figure 4A). Accuracy measures (sensitivity and specificity) were calculated using the RNA quantity values obtained by the NanoString nCounter as follows: (1) an RNA-quantity threshold was set up to 5-fold the maximum value of the control samples; (2) the sensitivity was calculated as (TP/P) × 100, where TP (true positives) is the number of OSCA samples showing an RNA quantity exceeding the threshold value, and P (positives) is the total number of OSCA samples screened; and (3) the specificity was calculated as (TN/N) × 100, where TN (true negatives) is the number of control samples showing an RNA quantity below the threshold value, and N (negatives) is the total number of control samples screened. Given the selected threshold value, the specificity was 100% for any gene.

## Data Availability

OSCA TCGA data are available at https://www.cancer.gov/tcga (accessed on 1 January 2019) GTex data on normal tissues are available at https://gtexportal.org/ (accessed on 1 January 2019). All data are available upon reasonable request to the corresponding author.

## Supplementary Materials

The following supporting information can be downloaded at: https://www.mdpi.com/article/10.3390/cancers14153764/s1, Table S1: Ranks of the 41 OSCA biomarkers from TCGA and GTEx, Figure S1: Gene Ontology enrichment analysis of 41 OSCA biomarkers.
