# Supplementary material for: Genome-Wide Identification and Validation of Gene Expression Biomarkers in the Diagnosis of Ovarian Serous Cystadenocarcinoma
Source: Cancers (Basel). 2022 Aug 2;14(15):3764. doi: 10.3390/cancers14153764 (PMC9367275; doi:10.3390/cancers14153764)
Supplement: Supplementary file 1 [file cancers-14-03764-s001.zip › Supplementary_Material_ proof_new_adg.pdf]

SUPPLEMENTARY MATERIAL

Supplementary Table S1. Ranks of the 41 OSCA biomarkers from TCGA and GTEx

GENE	OSCA Av. Exp. (Rank)	Control Av. Exp. (Rank)	GENE	OSCA Av. Exp. (Rank)	Control Av. Exp. (Rank)	GENE	OSCA Av. Exp. (Rank)	Control Av. Exp. (Rank)
**KRT8**	14.41 (55)	3.50 (9367)	**DSP**	13.03 (218)	1.41 (10898)	**ITGB4**	12.10 (593)	2.56 (9600)
**WFDC2**	14.21 (70)	2.48 (9667)	**PAX8**	12.87 (262)	0.82 (11765)	**KLK7**	12.06 (612)	0.07 (14788)
**MIF**	14.04 (80)	1.92 (10293)	**ELF3**	12.81 (285)	0.22 (13519)	**FOLR1**	11.93 (708)	0.18 (13729)
**KRT18**	13.95 (90)	6.00 (6855)	**EPCAM**	12.77 (297)	0.66 (12091)	**SOX17**	11.91 (720)	2.37 (9791)
**SLC34A2**	13.67 (123)	0.21 (13568)	**SPINT1**	12.62 (350)	1.42 (10878)	**LYPD1**	11.87 (759)	0.26 (13313)
**MSLN**	13.60 (128)	1.55 (10722)	**ST14**	12.50 (395)	0.42 (12703)	**PRSS8**	11.84 (783)	0.26 (13300)
**CLDN3**	13.54 (138)	0.27 (13262)	**CP**	12.50 (402)	0.60 (12234)	**S100A1**	11.79 (817)	0.21 (13587)
**MUC16**	13.53 (140)	0.06 (14977)	**PTGS1**	12.47 (412)	1.08 (11335)	**CLDN7**	11.74 (855)	0.47 (12561)
**HMGA1**	13.41 (159)	2.39 (9775)	**ADGRG1**	12.34 (461)	1.57 (10696)	**THSD4**	11.73 (873)	1.17 (11191)
**CLDN4**	13.29 (172)	0.29 (13194)	**TACSTD2**	12.33 (466)	0.93 (11580)	**ESRP1**	11.73 (875)	0.12 (14215)
**KRT7**	13.22 (186)	0.44 (12643)	**CDH1**	12.26 (498)	1.20 (11147)	**MYH14**	11.71 (883)	0.17 (13832)
**UCP2**	13.21 (187)	1.81 (10438)	**SORT1**	12.22 (524)	3.35 (8853)	**KLK6**	11.62 (957)	0.40 (12780)
**SCNN1A**	13.06 (210)	1.75 (10497)	**FAM83H**	12.17 (542)	0.28 (13225)	**ATP6V1B1**	11.59 (993)	0.97 (11504)
**MAL2**	13.06 (213)	0.20 (13663)	**CELSR2**	12.14 (566)	0.73 (11936)			

Average gene expression values (Av. Exp.) are indicated using the measure units originally reported in TCGA, i.e. TPM for OSCA samples, and in GTEx, i.e. RPKM for control healthy ovarian tissues. The rank indicates the position that the gene occupies in the lists of 18734 genes ordered from the highest to the lowest average gene expression value for OSCA samples and control samples separately.

Supplementary Table S2. Information on patients affected by the OSCA samples analyzed by the NanoString nCounter technology.

**Median age**		year (range)
		60.6 (40-87)
**FIGO stage**		number (%)
	I	5 (11.9)
	II	8 (19.1)
	III	25 (59.5)
	IV	4 (9.5)
**pN**		number (%)
	0	35 (83.3)
	1	7 (16.7)
**pM**		number (%)
	0	38 (90.5)
	1	4 (9.5)
**Grade**		number (%)
	1	0 (0)
	2	10 (23.8)
	3	32 (76.2)

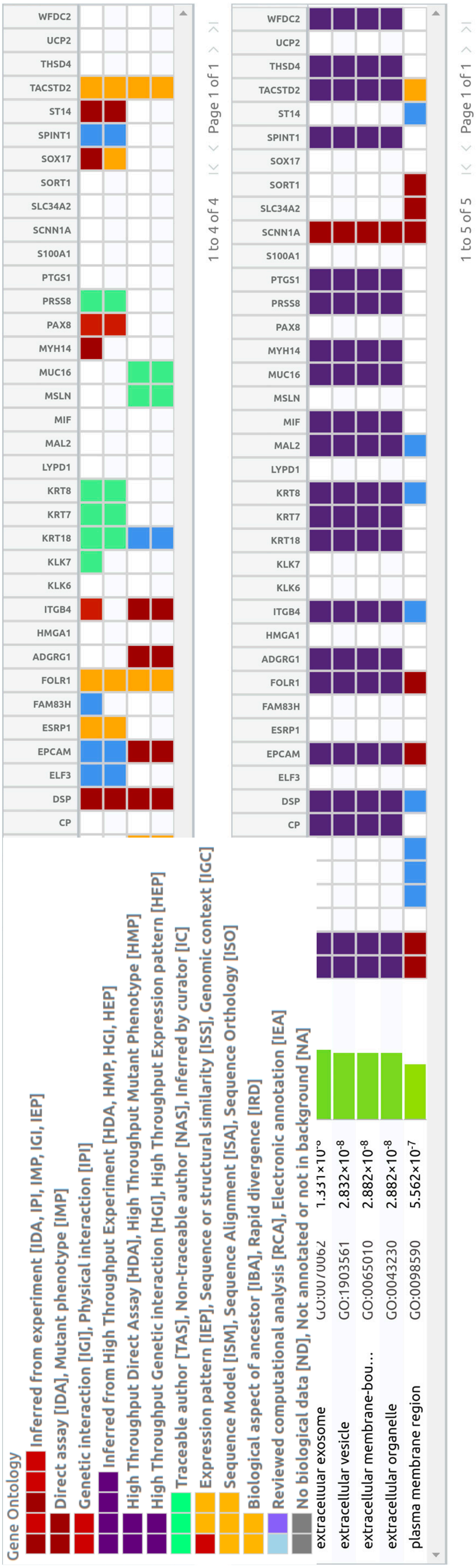

Supplementary Figure S1. Gene Ontology enrichment analysis of 41 OSCA biomarkers.

Output of the g:Profiler tool reporting statistically significant Gene Ontology terms that were over-represented in the 41 OSCA gene-expression biomarkers.
